# Comparison of trace elements in peripheral blood and bone marrow of newly diagnosed multiple myeloma patients

**DOI:** 10.1007/s10238-024-01349-5

**Published:** 2024-04-17

**Authors:** Ayse Nilgun Kul, Bahar Ozturk Kurt

**Affiliations:** 1 Department of Hematology, Kartal Dr. Lütfi Kırdar City Hospital, Cevizli, D-100 Guney Yanyol, Cevizli Mevkii No:47, 34865 Kartal/Istanbul, Turkey; 2 Department of Biophysics, Cerrahpaşa Faculty of Medicine, Istanbul University-Cerrahpaşa, Istanbul, Turkey

**Keywords:** Cancer, Multiple myeloma, Trace element

## Abstract

Trace elements are essential micronutrients for the human body. Their roles are indispensable, as they are involved in a wide range of vital biological processes. In this study, we aimed to evaluate alterations in trace elements in the blood and bone marrow serum of patients with newly diagnosed multiple myeloma (NMM). The levels of zinc (Zn), copper (Cu), iron (Fe), manganese (Mn), magnesium (Mg), selenium (Se), arsenic (As), boron (B), nickel (Ni), silicon (Si) and chromium (Cr) were analyzed in the venous blood samples of the patient group comprising 70 patients with NMM (41 males and 29 females) and compared to those in the control group comprising 30 individuals (18 males and 12 females). In addition, trace element levels were analyzed in bone marrow samples from the patient group. Blood and bone marrow serum levels were quantified using inductively coupled plasma optical emission spectrometry. When the blood samples of the patient and control groups were compared: Zn (*p* = 0.011), Fe (*p* = 0.008), Mn (*p* = 0.046), Se (*p* < 0.001), As (*p* < 0.001), Ni (*p* < 0.001) and Cr (*p* < 0.001) levels were significantly higher in the patient group than in the control group. Higher Zn, Fe, Mn, Se, As, Ni and Cr levels in the NMM patients suggest that alterations of trace elements could be predisposing factor that initiates the malignant process. The relationship between malignancies and trace elements is crucial for the development of adjuvant therapy strategies and preventive medicine and as biomarkers for cancer diagnosis. Therefore, there is a need for studies examining the relationship between hematological malignancies and trace elements.

## Introduction

Trace elements (TEs) [1], also known as micronutrients or essential minerals, are chemical elements required by the human body in very small amounts for proper physiological function. Although they are needed in small quantities, their roles are indispensable, as they are involved in a wide range of vital biological processes. These processes include enzymatic reactions, cellular signaling, immune response, deoxyribonucleic acid (DNA) synthesis and repair, antioxidant defense and the maintenance of structural integrity in tissues and bones. Common trace minerals include zinc, selenium, copper, iron, manganese, chromium and molybdenum. Despite their minimal presence in the body, TEs play a critical role in maintaining health and preventing various diseases. Imbalances in the levels of these elements can lead to a variety of health problems, including metabolic disorders, neurodegenerative diseases and immune dysfunction. Their intricate interactions with cellular pathways make them interesting candidates for research into their potential role in cancer development, progression and treatment.

Cancer is a complex and multifactorial disease characterized by uncontrolled cell growth and division leading to the formation of malignant tumors. It results from genetic mutations and epigenetic changes that disrupt the normal regulatory mechanisms that control cell proliferation and differentiation. Cancer development involves a dynamic interplay between genetic predisposition, environmental factors, lifestyle choices and aging. The transformation of normal cells into cancer cells involves a number of hallmarks, including sustained proliferative signaling, growth suppressor evasion, resistance to cell death, replicative immortality, angiogenesis and invasion and metastasis. These complex processes are influenced by a multitude of factors, making cancer a heterogeneous disease with different manifestations in different tissues and individuals. Alterations in the distribution of TEs in tissues and serum have been reported in patients with various cancers [2]. Estimating trace element levels in different tissues of the body can provide different malignant tumor profiles depending on the type and location of the cancer [3]. While numerous studies have investigated the association between TEs and solid organ cancers, such as breast, lung, colorectal and pancreatic cancers, there exists a scarcity of research investigating the association between TEs and hematological malignancies. However, their role in carcinogenesis remains unclear.

Multiple myeloma (MM), a malignant plasma cell disorder characterized by the uncontrolled proliferation of plasma cells in the bone marrow, remains a major challenge in the field of hematological malignancies [4]. MM is a rare type of hematological cancer that accounts for approximately 1% of all cancers [5]. The disease usually occurs in the elderly, with a current global incidence of 160,000 and mortality rate of 106,000 [3]. The etiology of MM has not been completely understood, but many factors such as genetic predisposition, karyotype abnormalities, familial causes, ionizing radiation, chronic diseases, chronic antigenic stimulation, autoimmune diseases, viruses, cytokines, exposure to chemical agents and working conditions have been proposed in the literature [6]. This incurable disease with unclear etiopathogenesis, continues to attract the attention of many researchers.

Despite advances in treatment methods, the inability to completely treat myeloma has led to the search for noninvasive and inexpensive alternatives, such as nutritional supplements [3]. Therefore, this article embarks on an exploratory journey into the intricate relationship between multiple myeloma and essential elements, looking at current research findings, potential mechanisms and implications for clinical practice. The primary objective of this study was to investigate the association between the levels of trace elements such as zinc (Zn), copper (Cu), iron (Fe), manganese (Mn), magnesium (Mg), selenium (Se), arsenic (As), boron (B), nickel (Ni), silicon (Si) and chromium (Cr) and changes in MM pathogenesis, thereby contributing to the identification of new targets for diagnosis and treatment of the disease.

## Materials and methods

### Patient population

All patients older than 18 years of age who were admitted to Kartal Dr. Lütfi Kırdar City Hospital, Hematology Outpatient Clinic, Istanbul, Turkey, between December 2021 and December 2022 and with a recent diagnosis of MM confirmed by bone marrow aspiration, biopsy, laboratory and genetic tests according to the myeloma diagnostic criteria revised by the International Myeloma Study Group (IMWG) diagnostic criteria were included in our study. Patients with treatment indications for recurrent or refractory disease and patients with a history of gammopathy of undetermined significance, smoldering myeloma or solitary plasmacytoma were excluded. A total of 70 patients, 41 men and 29 women were included the study. The control group consisted of 30 individuals, 18 men and 12 women, aged between 18 and 84 years. Informed consent was submitted by all subjects when they were enrolled. Patients with a history of chemotherapy and malignancy, taking nutritional supplements within 6 months before diagnosis, with chronic gastrointestinal disease requiring treatment, and taking nutritional supplements for any reason were excluded. Also, patients with a history of blood transfusion excluded that it may affect serum iron levels. Trace element levels were studied and compared in the venous blood samples of the patients and control groups. The TEs studied were Zn, Cu, Fe, Mg, Mn, Se, As, B, Ni, Si and Cr. In addition, trace element levels were compared by grouping the patients according to age, gender and genetic risk factors. Patients were divided into age groups of under 60 years and over 60 years, and genetic risk groups of standard and high risk. According to revised international scoring system (R-ISS), patients with deletion 17p, t(4,14) and t(14,16) were considered high risk, whereas patients without these genetic risk factors were considered standard risk. The present study protocol was reviewed and approved by the Clinical Research Ethics Committee of Kartal Dr. Lütfi Kırdar City Hospital (decision date: 09.06.2021, decision no.: 2021/514/203/1) and was conducted in agreement with the ethical standards specified in the Declaration of Helsinki.

### Parameters and procedures

Our study was designed in accordance with the ethical guidelines of the Declaration of Helsinki, revised in 2013 by the World Medical Association. 2 cc venous blood samples from newly diagnosed multiple myeloma patients and 2 cc bone marrow samples from bone marrow biopsy were collected in BD Vacutainer^®^SST™II Advance serum gel tubes. Blood samples were collected before any treatment was administered and centrifuged at 3000 rpm for 15 min. Red blood cell fraction and plasma were separated and transferred into fresh tubes and used for further analysis. Then, the sera were stored at − 70 °C until analysis. Serum TEs were quantified via an inductively coupled plasma optical emission spectrometry, the ICAP 6000 (ICP-OES by Thermo, UK). Before the analysis of TEs, serum samples were filtered, and 1 ml of serum was diluted in 10 cc of 0.3% HNO_3_ and vortexed. Standard concentrations were prepared using a 1000 ppm stock solution obtained from the chemistry laboratory for standard calibration curves. A spectrophotometric method for the analysis of all elements had a linear calibration curve of 50 and 100 ppb for Cr, Mn and Se standards, 500 and 1000 ppb for Cu, Fe, Mg and Zn standards, and 50, 100 and 1000 ppb for As, B and Si standards, respectively. The absorbances of Cr, Mn, Se, Cu, Fe, Mg, Zn, As, B and Si were read at 267.7, 257.6, 196.0, 324.7, 259.9, 285.2, 206.2, 189.0, 249.7 and 251.6 nm, respectively. The correlation coefficient of the lines was approximately 1 (0.999 for most analytes). Multiple wavelengths were used for each element to confirm quantitative results, but only results at one wavelength were reported for each element. Calibration standards and samples for whole blood analysis were monitored in radial mode at all wavelengths, whereas analysis of serum samples at all wavelengths was performed in both observation modes (axial and radial).

### Statistical analysis

All analyses were performed using IBM SPSS Statistics for Windows, version 25.0 (IBM Corp., Armonk, NY, USA). The Shapiro–Wilk test was used to determine whether variables were normally distributed. Data were presented as mean ± standard deviation or median (1st quartile–3rd quartile) for continuous variables, depending on the normality of the distribution, and as frequency (percentage) for categorical variables. More detailed descriptive statistics (including confidence interval, minimum, maximum, 5th percentile and 95th percentile) were given for trace element levels in bone marrow samples. Normally distributed variables were analyzed with Student’s *t*-test. Non-normally distributed variables were analyzed with Mann–Whitney *U* test. Categorical variables were analyzed with Chi-square tests or the Fisher’s exact test. *p* < 0.05 values were considered statistically significant.

## Results

The study included 70 patients and 30 healthy controls. In the patient group, 58.57% were male and 41.43% were female, with a mean age of 63.86 years. The control group included patients with similar characteristics of sex, age and comorbidities (Table 1). In terms of demographic characteristics, there was no significant difference between the patient and control groups. In the patient group, IGG kappa was the most common malignant clone with 37.14% (*n* = 26), and IGG lambda was the second most common with 25.71% (*n* = 18). While 71.43% (*n* = 50) of the patients had high risk genetic characteristics, 28.57% (*n* = 20) had standard risk genetic characteristics (Table 2).

**Table 1:** Demographic characteristics of the patient and control groups

	Groups		*p*
	Patients (*n* = 70)	Controls (*n* = 30)	
Age	63.86 ± 11.20	65.83 ± 8.36	0.333
Sex			
Male	41 (58.57%)	18 (60.00%)	1.000
Female	29 (41.43%)	12 (40.00%)	
Comorbidities			
Diabetes mellitus	15 (21.43%)	6 (20.00%)	1.000
Hypertension	29 (41.43%)	10 (33.33%)	0.591
Ischemic heart disease	11 (15.71%)	6 (20.00%)	0.816
Chronic obstructive pulmonary disease	6 (8.57%)	2 (6.67%)	1.000
Other	3 (4.29%)	1 (3.33%)	1.000

Data are given as mean ± standard deviation or median (1st quartile–3rd quartile) for continuous variables according to normality of distribution and as frequency (percentage) for categorical variables

**Table 2:** Characteristics of myeloma patients

Age	63.86 ± 11.20
Sex	
Male	41 (58.57%)
Female	29 (41.43%)
Comorbidities	
Diabetes mellitus	15 (21.43%)
Hypertension	29 (41.43%)
Ischemic heart disease	11 (15.71%)
Chronic obstructive pulmonary disease	6 (8.57%)
Other	3 (4.29%)
Type of myeloma	
IgA kappa	7 (10.00%)
IgA lambda	12 (17.14%)
IgG kappa	26 (37.14%)
IgG lambda	18 (25.71%)
Kappa	2 (2.86%)
Lambda	4 (5.71%)
Genetic risk	
Standard	50 (71.43%)
High	20 (28.57%)

The results of Zn, Cu, Fe, Mg, Mn, Se, As, B, Ni, Si and Cr in the sera of patients and control groups are given in µg/dl (microgram/deciliter). The levels of Zn, Fe, Mn, Se, As, Ni and Cr were significantly higher in the patient group compared to the control group (*p* < 0.001) (Table 3). While Se levels were 28.55 ± 10.62 µg/dl in the patient group and 14.27 ± 2.05 µg/dl in the control group, the others were as follows: As, 61.70 (47.20–73.20) µg/dl and 0.28 (0.24–0.30) µg/dl; Ni, 20.60 (16.00–29.70) µg/dl and 0.15 (0.07–0.20) µg/dl; Cr, 4.55 (3.80–5.20) µg/dl and 2.06 (2.03–2.13) µg/dl; Zn, 117.33 ± 39.23 µg/dl and 100.99 ± 23.27 µg/dl; Fe, 166.95 (104.40–274.20) and 150.18 (65.32–165.17) µg/dl; Mn, 1.10 (0.90–1.48) µg/dl and 1.04 (0.88–1.12) µg/dl. Mg and Si levels were significantly lower in the patient group compared to the control group (*p* < 0.001). No significant differences were found between the groups for Cu and B (Table 3). When the serum levels of TEs in the patient group were evaluated based on intra-group comparisons according to age, sex and genetic characteristics, no statistically significant difference was found between the levels measured in patients under 60 (*n* = 25) and over 60 (*n* = 45) years of age. When serum levels of TEs in the patient group were evaluated based on intra-group comparisons according to age, only Se was found to be statistically significant. Similarly, B levels were found to be significant when compared in terms of genetic risk. There were no statistical differences in terms of other parameters or sex (Table 4, 5, 6).

**Table 3:** Trace element levels in blood samples by group

	Group		*p*
	Patient	Control	
Zinc, µg/dl	117.33 ± 39.23^a^	100.99 ± 23.27	0.011
Copper, µg/dl	102.85 (88.70–123.10)	122.18 (67.88–132.67)	0.958
Iron, µg/dl	166.95 (104.40–274.20)^b^	150.18 (65.32–165.17)	0.008
Magnesium, µg/dl	1530 (1323–1768)^c^	2183 (1972–2501)	< 0.001
Manganese, µg/dl	1.10 (0.90–1.48)^a^	1.04 (0.88–1.12)	0.046
Selenium, µg/dl	28.55 ± 10.62^c^	14.27 ± 2.05	< 0.001
Arsenic, µg/dl	61.70 (47.20–73.20)^c^	0.28 (0.24–0.30)	< 0.001
Boron, µg/dl	9.25 (5.20–14.40)	7.55 (4.80–18.00)	0.525
Nickel, µg/dl	20.60 (16.00–29.70)^c^	0.15 (0.07–0.20)	< 0.001
Silicon, µg/dl	104.95 (71.80–188.80)^c^	966.00 (721.00–1101.00)	< 0.001
Chromium, µg/dl	4.55 (3.80–5.20)^c^	2.06 (2.03–2.13)	< 0.001

Data are given as mean ± standard deviation or median (1st quartile–3rd quartile) for continuous variables according to normality of distribution and as frequency (percentage) for categorical variables

^a^*p* < 0.05, ^b^*p* < 0.01 and ^c^*p *< 0.001 vs. control

**Table 4:** Summary of trace element levels in blood samples in the patients group by age

	Age groups		*p*
	< 60 (*n* = 25)	≥ 60 (*n* = 45)	
Zinc, µg/dl	115.07 ± 43.53	118.59 ± 37.09	0.722
Copper, µg/dl	100.2 (86.7–119.4)	106.8 (89.6–126.1)	0.408
Iron, µg/dl	167.8 (123.3–337.3)	160.6 (104.4–273.8)	0.646
Magnesium, µg/dl	1526 (1362–1647)	1548 (1323–1768)	0.976
Manganese, µg/dl	1.1 (0.8–1.4)	1.1 (0.9–1.5)	0.240
Selenium, µg/dl	24.93 ± 11.71^a^	30.56 ± 9.51	0.033
Arsenic, µg/dl	63.4 (49.1–73.6)	58.4 (47.2–69.3)	0.633
Boron, µg/dl	9.2 (5.4–14.3)	9.3 (5.2–14.4)	1.000
Nickel, µg/dl	24.2 (19.4–29.3)	20.0 (15.4–29.7)	0.300
Silicon, µg/dl	102.1 (71.8–188.8)	105.2 (72.1–178.8)	0.917
Chromium, µg/dl	4.4 (3.9–5.2)	4.7 (3.8–5.2)	0.560

Data are given as mean ± standard deviation or median (1st quartile–3rd quartile) for continuous variables according to normality of distribution

^a^*p *< 0.05 vs. age ≥  60

**Table 5:** Summary of trace element levels in blood samples in the patient group by sex

	Sex		*p*
	Male (*n* = 41)	Female (*n* = 29)	
Zinc, µg/dl	119.80 ± 45.87	113.84 ± 27.67	0.501
Copper, µg/dl	97.1 (84.9–122.8)	116.2 (98.1–126.1)	0.051
Iron, µg/dl	186.3 (107.9–313.5)	154.1 (104.4–192.5)	0.236
Magnesium, µg/dl	1584 (1309–1866)	1483 (1361–1619)	0.260
Manganese, µg/dl	1.1 (0.8–1.4)	1.1 (1.0–1.5)	0.590
Selenium, µg/dl	29.50 ± 11.03	27.21 ± 10.05	0.378
Arsenic, µg/dl	61.5 (46.2–73.7)	62.1 (52.6–66.4)	0.981
Boron, µg/dl	8.97 (5.33–14.8)	10.0 (5.2–13.8)	0.986
Nickel, µg/dl	22.1 (16.9–29.7)	20.0 (15.4–27.2)	0.340
Silicon, µg/dl	107.0 (76.9–209.0)	102.7 (54.9–139.8)	0.319
Chromium, µg/dl	4.8 (4.0–5.5)	4.4 (3.6–4.9)	0.134

Data are given as mean ± standard deviation or median (1st quartile–3rd quartile) for continuous variables according to normality of distribution

**Table 6:** Summary of trace element levels in blood samples in the patient group by genetic risk

	Genetic risk		*p*
	Standard (*n* = 50)	High (*n* = 20)	
Zinc, µg/dl	114.95 ± 40.52	123.29 ± 36.11	0.426
Copper, µg/dl	100.00 (89.60–122.00)	117.45 (83.95–140.85)	0.304
Iron, µg/dl	175.70 (122.10–299.90)	136.25 (91.35–214.15)	0.232
Magnesium, µg/dl	1492.0 (1323.0–1654.0)	1605.5 (1335.5–2002.5)	0.298
Manganese, µg/dl	1.10 (0.90–1.50)	1.10 (0.90–1.44)	0.789
Selenium, µg/dl	28.57 ± 10.53	28.51 ± 11.10	0.983
Arsenic, µg/dl	60.95 (46.20–73.60)	61.85 (47.45–66.35)	0.891
Boron, µg/dl	9.97 (5.40–15.00)^a^	7.23 (3.35–10.20)	0.047
Nickel, µg/dl	20.60 (16.00–30.00)	20.55 (16.10–25.85)	0.658
Silicon, µg/dl	110.85 (72.10–188.80)	99.35 (60.20–196.65)	0.668
Chromium, µg/dl	4.80 (4.00–5.30)	4.40 (3.65–4.90)	0.249

Data are given as mean ± standard deviation or median (1st quartile–3rd quartile) for continuous variables according to normality of distribution

^a^*p* < 0.05 vs. high genetic risk group

The bone marrow samples were analyzed using the same methods as the venous blood samples. A voluntary control group could not be established due to the difficulty of the bone marrow biopsy procedure. Bone marrow samples obtained from the patient group at the time of diagnosis were used. As there were no previous studies in the literature on bone marrow TEs, no data could be obtained. Our data are the first study to report bone marrow trace element levels (Table 7). The results were given in µg/dl. Bone marrow trace element levels were as follows Zn; 168.99 ± 55.41 µg/dl, Cu; 161.26 ± 56.55 µg/dl, Fe; 570.13 ± 825.92 µg/dl, Mg; 2304.29 ± 679.27 µg/dl, Mn; 3.42 ± 0.76 µg/dl, Se; 103.03 ± 29.91 µg/dl, As; 53.29 ± 18.84 µg/dl, B; 4.90 ± 4.20 µg/dl, Ni; 101.18 ± 75.07 µg/dl, Si; 405.71 ± 446.24 µg/dl, Cr; 4.04 ± 2.78 µg/dl (Table 7).

**Table 7:** Summary of trace element levels in bone marrow samples

	Mean ± standard deviation (95% confidence interval)	Minimum	Percentile					Maximum
			5th	25th	50th	75th	95th	
Zinc, µg/dl	168.99 ± 55.41 (155.77–182.20)	17.72	66.50	144.90	172.75	195.30	267.00	295.80
Copper, µg/dl	161.26 ± 56.55 (147.77–174.74)	57.60	84.20	125.50	156.55	193.90	255.00	333.10
Iron, µg/dl	570.13 ± 825.92 (373.20–767.06)	103.00	123.00	177.80	354.30	527.10	2134.00	5836.00
Magnesium, µg/dl	2304.29 ± 679.27 (2142.32–2466.25)	1158.00	1352.00	1816.00	2111.50	2732.00	3557.00	4155.00
Manganese, µg/dl	3.42 ± 0.76 (3.24–3.60)	1.80	2.40	3.00	3.40	3.70	4.10	8.20
Selenium, µg/dl	103.03 ± 29.91 (95.90–110.16)	8.10	51.50	87.30	106.65	123.60	148.20	161.10
Arsenic, µg/dl	53.29 ± 18.84 (48.80–57.79)	15.70	30.00	39.60	52.25	63.40	94.40	109.60
Boron, µg/dl	4.90 ± 4.20 (3.90–5.90)	0.20	0.50	2.10	3.40	7.40	13.30	15.70
Nickel, µg/dl	101.18 ± 75.07 (83.28–119.08)	23.90	30.50	50.40	67.85	119.70	248.60	319.70
Silicon, µg/dl	405.71 ± 446.24 (299.31–512.11)	9.80	42.10	124.60	208.70	477.70	1452.00	1832.00
Chromium, µg/dl	4.04 ± 2.78 (3.38–4.71)	0.10	0.30	1.20	4.30	6.00	8.60	12.30

## Discussion

TEs, which constitute less than 0.01% of total body weight, are vital for basic cellular mechanisms. TEs have been reported to be associated with many fetal diseases including cancer. However, their exact role in carcinogenesis is still unknown. The majority of studies on carcinogenesis and TEs are based on solid tumors. These include lung, breast and pancreatic cancers and tumors of the gastrointestinal tract [7, 8]. Although there are a few studies on hematological cancers evaluating the relationship between TEs and acute leukemias and lymphomas, there are no data available for other hematological malignancies, particularly plasma cell disorders.

In one of these studies, Juloski et al. found that the Cu/Zn ratio in malignant tissue of colorectal cancer patients was higher than in healthy controls [9]. Similarly, a multicenter study conducted in 2019 revealed a notable disparity in trace element levels between pancreatic cancer patients with and without KRAS (Kirsten rat sarcoma virus) mutation [10–13]. Consequently, changes in trace element concentrations within the body may represent a potential predisposing factor for malignancy development. For instance, a 2010 study investigating the association between soil As levels and mortality rates in 29 Chinese regions with elevated leukemia mortality identified an inverse correlation between soil As levels and leukemia mortality [14].

At the end of our study, we realized that the results we obtained were compatible with and supportive of the literature data. Se, As, Ni and Cr, which we found to be significantly higher in the patient group compared to the control group, are among the 5 TEs reported to have carcinogenic effects on humans according to the study by IRAC (International Agency for Research on Cancer. Lyon. France) (*p* < 0.001) (Table 3). In the review on the effect of TEs on carcinogenesis published by Paolo Boffetta for IRAC in 1993, TEs with tumorigenesis effects on animals and humans were grouped. The trace element compounds that have a negative effect on humans in terms of malignancy were listed as arsenic and arsenic compounds, beryllium and beryllium compounds, cadmium and cadmium compounds, hexavalent chromium compounds and nickel compounds [15].

Se is a unique trace element that binds to a specific codon on mRNA and forms selenoproteins. There are studies suggesting that selenoproteins formed by this mechanism have anticarcinogenic effects as well as studies which conclude that they have carcinogenic effects. As of now, a consensus on this matter remains elusive [16–19]. In 2014, the SELECT study by Albanes et al. emphasized the close relationship of Se with high-grade prostate cancer and skin cancer in particular [20]. Two studies by Vincenti et al. in 2016 and 2018, which examined the relationship between multiple myeloma and Se, also mentioned that the frequency of multiple myeloma increased in patients who consumed water with high Se content [21, 22]. These results, support the view that TEs may play a role in the development of hematological malignancies, are consistent with the results of our study. In addition, Yuan et al., who conducted a Mendelian randomization study to examine the relationship between selenium and 22 site-specific cancers, did not find any association between selenium levels and MM [23].

As and its compounds are used in the treatment of acute promyelocytic leukemia and are known to have cytotoxic effects on cancer cells [24]. It is a logical paradox that As compounds used in cancer treatment are among the 5 elements identified as carcinogenic by IRAC. A 2021 study by Medda et al. examining the effect of As and its compounds on human biology, stated that the direction in which the As level affects the balance between proliferation and apoptosis determines the transformation, thereby offering a logical explanation to this paradoxical situation [25]. Another study that explores the paradox between carcinogenic and cytotoxic effect is the study by Khairul et al., which supports Medda’s findings [26]. In our study, the level of As in venous blood serum was significantly higher in the patient group compared to the control group. Since there was no study examining the relationship between As and MM, there were no data with which to compare our current results. However, higher serum As levels in MM patients suggest that As may play a role that change the balance to malignancy.

Ni was another trace element that we found to be significantly higher in the patient group than in the control group. Guo et al. reported that Ni damages DNA either by binding to DNA or via reactive oxygen radicals and is often involved in lung and nasal cancers [27]. Similarly, a 2020 review by Son emphasized that Ni increases the risk of malignancy due to its increasing industrial use in recent years and its cumulative effects [28]. DNA damage and chromosomal mutations which are thought to be the starting point for many cancers have also been defined in the genetic structure of MM patients. Therefore, it is a remarkable finding that Ni levels were significantly higher in our patient group.

Cr is a naturally occurring element found in soil, rock and living organisms. It can readily traverse cell membranes through nonspecific sulfate/phosphate channels and diffusion [29, 30]. Although it has been suggested that it is not carcinogenic, there is limited data on this subject. The IRAC and the EPA (U.S. Environmental Protection Agency) respectively classified Cr among Group I and Group A human carcinogens. It was found to have toxic effects, especially on respiratory, gastrointestinal and urogenital systems and skin. A 2009 animal study by Stout et al. concluded that Cr exhibits carcinogenic effects through oxidative stress, DNA damage by genomic instability and epigenetic modulation [31]. In a meta-analysis published by Cole et al. reviewing 49 epidemiologic studies, the relationship between Cr and cancer was investigated and no significant relationship was found with cancers other than gastric cancer [32]. Although occupational exposure to chromium has been reported to cause various malignancies, high Cr levels were found in our study group despite the absence of occupational exposure. While these findings are consistent with those of Yamamoto et al., this situation was associated with MM symptoms and general condition changes [5]. The lack to definitively establish or refute the association between Cr and MM suggests that further studies investigating the relationship between Cr and hematologic cancers are needed.

Zn regulates cell mediated immune functions and also acts as an antioxidant and anti-inflammatory agent. Disruption of zinc homeostasis leads to the formation of reactive oxygen species, which can result in alterations in immunity, aging and civilization diseases, also in cancer development. Many studies which investigates cancer and TEs relation often points toxicity of Zn on cancer cells. It is also recommended to be added to cancer treatment regimens in cancer patients. In particular, low serum Zn levels were mentioned in bladder and endometrial cancer patients [33, 34]. In fact, Bengtsson et al. emphasized that high Zn intake will improve the survival in breast cancer patients [35]. The use of Zn as an antioxidant to combat increased oxidative stress has been suggested to explain the decrease in Zn levels in MM [36, 37]. In contrast, Mohammed et al. were showed that Zn levels were increased in the MM patient group, similar to our findings [38]. Like Zn, Mn, is also participates in many cellular reactions. Because of the correlation between Mn levels and the cancer incidence, Mn supplementation is recommended according to European guidelines. Many studies report low levels of Mn in cancer patients, a review of 2023 by Golara A. et al. mentions the correlation between Mn levels and the incidence and course of cervical and ovarian cancer [39]. Although our study results seem to contradict the existing literature, tumor formation is unique to each cancer. It will be possible to reach a clear conclusion with the data to be obtained in the future.

Fe studies in MM patients strongly support the anemia of inflammation that is termed “anemia of chronic disease.” But, recently understood that impaired iron utilization due to increased pro-inflammatory cytokines formed by the nature of MM may be the reason. Consequently, this makes high Fe levels an expected finding [40, 41]. Epidemiological studies have suggested that Fe levels are positively associated with cancer risk [42]. It has been suggested that when blood is donated, the risk of cancer decreases with decreasing Fe levels [43], and when blood is transfused, the risk of cancer increases with increasing Fe levels [44]. Due to the pro-oxidative properties of Fe, Fe toxicity can occur because excess Fe can react with hydrogen peroxide via the Fenton reaction and produce ROS. MM cells are more sensitive to Fe toxicity than other cell types [45]. A previous study showed that serum ferritin levels were significantly elevated in patients with MM [46]. On the other hand, normal and low Fe levels are more common than Fe overload in MM patients [3]. The non-significant increase in Fe levels we observed supports this hypothesis. In terms of Fe, we also should point the study of Vanderwall et al. which underlines the dependence of malignant myeloma cells on an increased influx of Fe and suggested preclinical investigations into adjuvant therapy strategies to target this requirement. The induction of Fe deficiency in patients with MM, underscore the significance of the interplay between Fe and apoptosis [40, 41].

In our study, the low Mg levels in MM patients compared to the control group may suggest that Mg deficiency contributes to malignancy or that Mg deficiency occurs secondary since it is needed for vital functions of tumor burden. In a review published by Castiglioni and Maier, it has been emphasized that there is a dangerous threshold between the presence and deficiency of magnesium. It was stated that in vitro tumor cells require Mg for their vital functions and that tumorigenesis is reduced in Mg deficiency. At the same time, they introduced a dilemma by pointing out that Mg deficiency in healthy cells induces tumorigenesis [47]. As recommended by Castiglioni and Maier, further studies are needed on this subject.

Si is the second most common element in nature and many forms of silicon are resorbable, water soluble and bioavailable. Contact with Si is through environmental exposure and dietary intake [48]. There are no data on Si being carcinogenic, but it is noteworthy that Si levels were lower in the patient group than in the control group.

Although there was no significant difference between the patient and control groups in terms of Cu and B serum values. We would like to underline the study Valadbeigi et al. conducted a study that compared trace element levels in a patient cohort consisting of 32 ALL (acute lymphoblastic leukemia) and 16 AML (acute myeloid leukemia) patients–with a healthy control group comprising 36 individuals. The study revealed significantly reduced levels of Zn and Se, along with notably elevated levels of Cu in the serum of acute leukemia patients [49]. Serum Cu levels in lymphoma patients were shown to increase at relapse, while remission was linked to the fall of Cu levels back to normal ranges. For this reason, it was suggested that serum Cu levels may be a predictor of chemotherapy response in leukemia and malignant lymphoma [50, 51].

When compared by age, sex and genetic risk group, TE levels were similar in both groups, suggesting that TE levels were not affected by age, sex and genetic risk characteristics. (Table 4,5,6).

The results of TEs in sera obtained from the bone marrow of MM patients are reported as mean and minimum/maximum levels (Table 7). Since bone marrow biopsy is an uncomfortable and invasive procedure, a healthy volunteer group could not be formed and this might appear to be a limitation; however, our study is the first to include data on bone marrow trace element levels in MM patients. Due to the lack of data in the current literature, it is not possible to comment on the results. Therefore, we believe that our study can provide a baseline for future studies.

In conclusion, higher levels of Zn, Fe, Mn, Se, As, Ni and Cr in multiple myeloma patients than in controls suggest that there may be a relationship between deterioration of the balance in this direction and the development of cancer. The malignancy and TEs relevance holds significant importance for the development of adjuvant therapy strategies and preventive medicine. It may also be used as an option for disease monitoring, early detection of relapse and as a biomarker for cancer diagnosis. For this reason, further studies involving large patient populations are required.
